# Mechanistic Analysis of Chemically Diverse Bromodomain-4 Inhibitors Using Balanced QSAR Analysis and Supported by X-ray Resolved Crystal Structures

**DOI:** 10.3390/ph15060745

**Published:** 2022-06-14

**Authors:** Magdi E. A. Zaki, Sami A. Al-Hussain, Aamal A. Al-Mutairi, Vijay H. Masand, Abdul Samad, Rahul D. Jawarkar

**Affiliations:** 1 Department of Chemistry, Faculty of Science, Imam Mohammad Ibn Saud Islamic University, Riyadh 13318, Saudi Arabia; sahussain@imamu.edu.sa (S.A.A.-H.); aamutairi@imamu.edu.sa (A.A.A.-M.); 2 Department of Chemistry, Vidya Bharati Mahavidyalaya, Amravati 444602, India; 3 Department of Pharmaceutical Chemistry, Faculty of Pharmacy, Tishk International University, Erbil 44001, Iraq; abdul.samad@tiu.edu.iq; 4 Department of Medicinal Chemistry, Dr. Rajendra Gode Institute of Pharmacy, University-Mardi Road, Amravati 444901, India; rahuljawarkar@gmail.com

**Keywords:** QSAR, BRD-4, pharmacophoric features, X-ray

## Abstract

Bromodomain-4 (BRD-4) is a key enzyme in post-translational modifications, transcriptional activation, and many other cellular processes. Its inhibitors find their therapeutic usage in cancer, acute heart failure, and inflammation to name a few. In the present study, a dataset of 980 molecules with a significant diversity of structural scaffolds and composition was selected to develop a balanced QSAR model possessing high predictive capability and mechanistic interpretation. The model was built as per the OECD (Organisation for Economic Co-operation and Development) guidelines and fulfills the endorsed threshold values for different validation parameters (R^2^_tr_ = 0.76, Q^2^_LMO_ = 0.76, and R^2^_ex_ = 0.76). The present QSAR analysis identified that anti-BRD-4 activity is associated with structural characters such as the presence of saturated carbocyclic rings, the occurrence of carbon atoms near the center of mass of a molecule, and a specific combination of planer or aromatic nitrogen with ring carbon, donor, and acceptor atoms. The outcomes of the present analysis are also supported by X-ray-resolved crystal structures of compounds with BRD-4. Thus, the QSAR model effectively captured salient as well as unreported hidden pharmacophoric features. Therefore, the present study successfully identified valuable novel pharmacophoric features, which could be beneficial for the future optimization of lead/hit compounds for anti-BRD-4 activity.

## 1. Introduction

Cancer and heart failure are major causes of mortality [1], health complications, and social and economic problems for millions of people around the globe. Researchers have identified different chemotherapeutic methods to minimise heart failure as well as the onset, growth, and survival of cancer cells [1]. However, different serious health issues initiated or echoed by different anti-cancer and cardiac drugs are of great concerns. Therefore, the quest for a harmless and effective anti-cancer and cardiac drug is an important goal for the research and development laboratories of pharmaceutical companies and academic institutions. For this, researchers generally prefer to inhibit any irregularity occurring during a vital cellular process. A good number of recent studies have confirmed that reversible lysine acetylation (RAL) is a dynamic process responsible for protein post-translational modifications, transcriptional activation, and other cellular processes [2,3,4,5,6,7,8,9,10,11]. Therefore, any anomaly with RAL could lead to the initiation of malignancy or its survival [7,12]. RAL is regulated by three types of epigenetic regulatory proteins [12,13]: (1) histone acetyltransferases (HATs) acetylate lysine, (2) Histone deacetylases (HDACs), and (3) bromodomain (BRD) family of proteins. HATs are responsible for acetylation of lysine residues on histone tails and thereby behave as “writers”, whereas the reverse is true for HDACs and sirtuins, which work as “erasers” that are accountable for the elimination of the acetyl group from acetylated lysine (KAc) [14]. The Bromodomain and Extra-terminal (BET) family selectively recognises and links with acetylated lysine residues in histones H3 and H4 [15,16,17]; thus, they function as “readers”. The BET proteins, viz., BRD2, BRD3, and BRD-4, and bromodomain testis-specific protein (BRDT) are widely recognised as druggable target proteins for regulating cellular epigenetics [15]. Therefore, intruding interactions between BET proteins and acetylated lysine have attracted many researchers to develop better therapeutics for various human diseases including cancer, acute heart failure, and inflammation [2,3,4,5,6,7,8,9,10,11].

**Figure 1:**
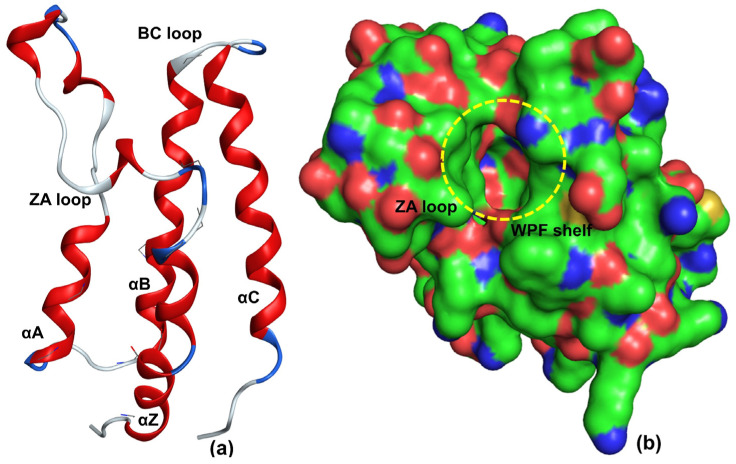
X-ray resolved structure of BRD-4 using pdb 5UVT (**a**) without molecular surface and (**b**) with molecular surface (green: carbon; red: oxygen; blue: nitrogen).

BRD-4, also called mitotic chromosomal-associated protein (MCAP), Fshrg4, or Hunk1, is ubiquitously expressed and plays a crucial role in a number of DNA-centered processes [15]. It is generally localised in the nucleus and regulates transcription by RNA polymerase II through a positive transcriptional elongation factor complex [15]. Structurally, it comprises two highly conserved N-terminal bromodomains (BD1 and BD2), an ET domain, and a C-terminal domain (CTD) [7]. Furthermore, BRD-4 contains a set of four helices: αZ, αA, αB, and αC. αZ and αA helices are connected through the ZA loop, whereas the BC loop connects the αB and αC helices [11,18]. Together, the four helices and the two loops create an active acetyl-lysine binding pocket (see Figure 1) [11,18]. The active site also consists of a hydrophobic WPF shelf (Trp81, Pro82, and Phe83), ZA loop, Tyr97, Asn140, and Met149 [11,18]. The majority of BRD-4 inhibitors compete with histone H4 to imitate the interactions with Tyr97 and Asn140 [3]. The WPF shelf is believed to play an important role in deciding the selectivity for BET bromodomains [6].

**Figure 2:**
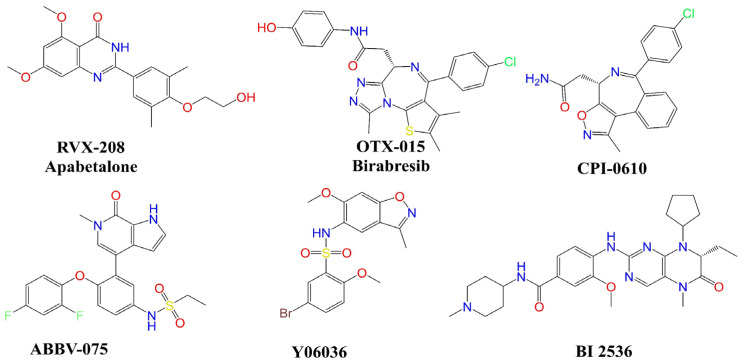
Chemical structures of selected BRD-4 inhibitors.

Recent studies indicate that the inhibition of BRD-4 is a good strategy, and a good number of BRD-4 inhibitors are in clinical or pre-clinical trials (see Figure 2) [2,19,20].

However, the quest for a safer and effective BRD-4 inhibitor with an optimum ADMET (Absorption, Distribution, Metabolism, Excretion, and Toxicity) profile with a retention of potency is still in progress. For this, it is essential to know the prominent and concealed pharmacophoric features associated with BRD-4 inhibitors. To achieve this goal, a good number of researchers have reported SAR (Structure Activity Relationships) and QSAR (Quantitative SAR) analyses of BRD-4 inhibitors. Tahir et al. [21] developed a CoMSIA (3D-QSAR) model with an R^2^_tr_ (coefficient of determination) = 0.982 and R^2^_cv_ (or Q^2^_loo_) (cross-validated coefficient of determination for leave-one-out) = 0.500 for a dataset of 60 quinolinone and quinazolinone derivatives as BRD-4 inhibitors. Tong et al. [22] reported four 3D-QSAR models possessing R^2^_tr_ = 0.912 to 0.963 and R^2^_cv_ = 0.574 to 0.759 for the BRD-4 inhibitory activity of 4,5-dihydro-[1,2,4]triazolo[4,3-f]pteridine derivatives. Obadawo and co-workers [23] performed QSAR modelling (R^2^_tr_ = 0.93 and R^2^_cv_ = 0.70) for 40 different substituted 4-Phenylisoquinolinones as potent BET bromodomain (BRD-4-BD1) inhibitors. Speck-Planche and Scotti [6] performed multi-target QSAR for bromodomain inhibitors using linear discriminant analysis and artificial neural networks. Their binary classification (active/inactive), which is based on a fragment-based topological approach, and analysis led to the identification of a good number of pharmacophoric features. However, the fragment-based topological approach involved the use of SMILES of molecules and thereby lacks the inclusion of 3D information. Thus, even though these studies are successful in identifying easily visible pharmacophoric features, they are based on small datasets with limited variations in structures, binary classification, lack thorough validation and general applicability, and provide partial mechanistic interpretations.

A literature survey reveals that BRD-4 inhibitors possess structural isomerism (positional, chain, etc.), variations in central scaffolds, and their chemical space is very broad [6,7]; therefore, many concealed or hidden correlations of pharmacophoric features cannot be identified by visual inspection [24]. In such a situation, there is a need to accomplish thorough QSAR analysis using a larger dataset of BRD-4 inhibitors. In the present work, we have performed QSAR analysis of 980 structurally diverse BRD-4 inhibitors. The developed QSAR model possesses a balance of excellent predictive ability with in-depth mechanistic interpretations, which are reinforced by reported X-ray-resolved structures of BRD-4 inhibitors with the target enzyme.

## 2. Results

The present QSAR analysis is based on a dataset covering a broad chemical space and data range owing to the inclusion of structurally diverse compounds with experimentally measured IC_50_ in the range of 1 nM to 15 µM. Consequently, this helped us in developing an appropriately validated genetic algorithm multi-linear regression (GA-MLR) model for gathering or extending exhaustive information about the pharmacophoric traits that govern the desired bio-activity (Descriptive QSAR) and also possessing acceptable external predictive capability (Predictive QSAR) [25,26,27]. The seven variable-based GA-MLR QSAR model (see model-A), along with selected internal and external validation parameters (see Supplementary Material for additional parameters), is as follows.

**Model-A:** pIC_50_ = 4.27 (±0.156) + 0.093 (±0.017) * fsp3CringC2B + 0.108 (±0.014) * com_C_4A + 0.391 (±0.066) * Saturated_Carbo_Rings + 0.428 (±0.036) * fsulfonSaroC8B + 0.625 (±0.059) * flipoacc3B + 0.921 (±0.067) * fsp3OaroN6B—0.367 (±0.063) * fplaNN4B.

**Validation of Model-A:** Method of splitting = Random, No. of descriptors = 7, N_training_ = 785, N_test_ = 195, R^2^_tr_ = 0.762, R^2^_adj._ = 0.760, RMSE_tr_ = 0.389, MAE_tr_ = 0.326, CCC_tr_ = 0.865, s = 0.391, F = 355.446, R^2^_cv_ (Q^2^loo) = 0.757, RMSE_cv_ = 0.393, MAE_cv_ = 0.329, CCC_cv_ = 0.862, Q^2^_LMO_ = 0.756, R^2^_Yscr_ = 0.009, Q^2^_Yscr_ = −0.012, RMSE_ex_ = 0.392, MAE_ex_ = 0.323, R^2^_ex_ = 0.762, Q^2^-F^1^ = 0.762, Q^2^-F^2^ = 0.760, Q^2^-F^3^ = 0.758, CCC_ex_ = 0.860.

**Figure 3:**
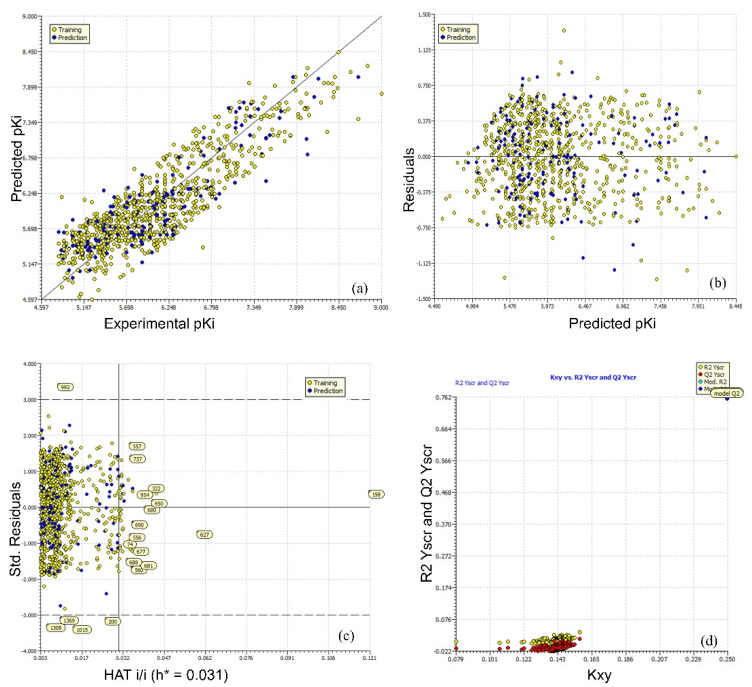
Different graphs associated with the model: (**a**) experimental vs. predicted pIC_50_ (the solid line represents the regression line); (**b**) experimental vs. residuals values; (**c**) Williams plot for applicability domain (the vertical solid line represents h * = 0.031 and horizontal dashed lines represent the upper and lower boundaries for standard residuals); (**d**) Y-randomization.

A multitude of statistical validation parameters and analysis of associated graphs has been recommended by different researchers to confirm the statistical robustness and external prediction ability of a QSAR model [28,29,30,31,32,33,34,35,36,37,38,39]. The same approach has been followed in the present work. A high value of R^2^_tr_, R^2^_adj._, R^2^_cv_ (Q^2^loo), R^2^_ex_, Q^2^-F^n^**,** CCC_ex_, etc., and a small value of LOF (lack-of-fit), RMSE_tr_, MAE_tr_, R^2^_Yscr_ (R^2^ for Y-scrambling), etc., along with different graphs (Figure 3a–d) related to model-A support the external predictive ability, statistical robustness, and point outs the lack of chancy correlation for model-A [28,29,30,31,32,33,34,35,36,37,38]. Moreover, the Williams plot [40,41,42,43,44] point outs that the majority of molecules (929 molecules) are within the applicability domain; thus, the model is statistically acceptable (see Figure 3b). The outliers with high leverage have been labeled in Figure 3b. Therefore, it fulfills all the Organisation for Economic Co-operation and Development (OECD) endorsed guidelines for generating a thriving QSAR model.

The descriptions of seven molecular descriptors constituting model-A have been tabulated in Table 1.

Interestingly, five molecular descriptors, viz., com_C_4A, fsp3CringC2B, flipoacc3B, fsulfonSaroC8B, and Saturated_Carbo_Rings, comprise the presence of different types of carbon atoms, which indicates the importance of carbon atoms in deciding BRD-4 inhibitory activity. The same is true for nitrogen, which is a part of three molecular descriptors, viz., flipoacc3B, fplaNN4B, and fsp3OaroN6B. Since, in general, the presence of carbon increases lipophilicity whereas nitrogen is attributed to significantly influence the pharmacological and hydrophilic profile, therefore, a balance of an appropriate number of carbons for lipophilicity and nitrogen is necessary to obtain adequate BRD-4 inhibitory activity. Of the seven descriptors in model-A, six have positive coefficients and only one has a negative coefficient. The effects of descriptors and their role in deciding the BRD-4 inhibitory profile have been discussed in more detail with relevant examples in the Discussion section.

## 3. Discussion

### Mechanistic Interpretation of QSAR Model

An appropriately validated relationship between prominent structural features or molecular descriptors of the molecules with the bioactivity enlarges knowledge about mechanism of action of molecules, reasons for their specificity, and pharmacophoric atoms/groups accountable for the desired bioactivity [20,26,39]. In the present analysis, although we have equated the IC_50_ values of different molecules in a relationship with a specific molecular descriptor (or feature), a synergistic or reverse effect of other molecular descriptors or unknown factors having a superseding influence in deciding the overall IC_50_ value of a molecule cannot be ignored. That is, a single molecular descriptor or feature neither decides nor completely explains the experimental IC_50_ value for such a large and structurally diverse set of molecules. In other words, the effective use of a validated QSAR model depends on the synergetic consideration of constituent molecular descriptors. The newly developed QSAR model-A comprises seven descriptors.

**Figure 4:**
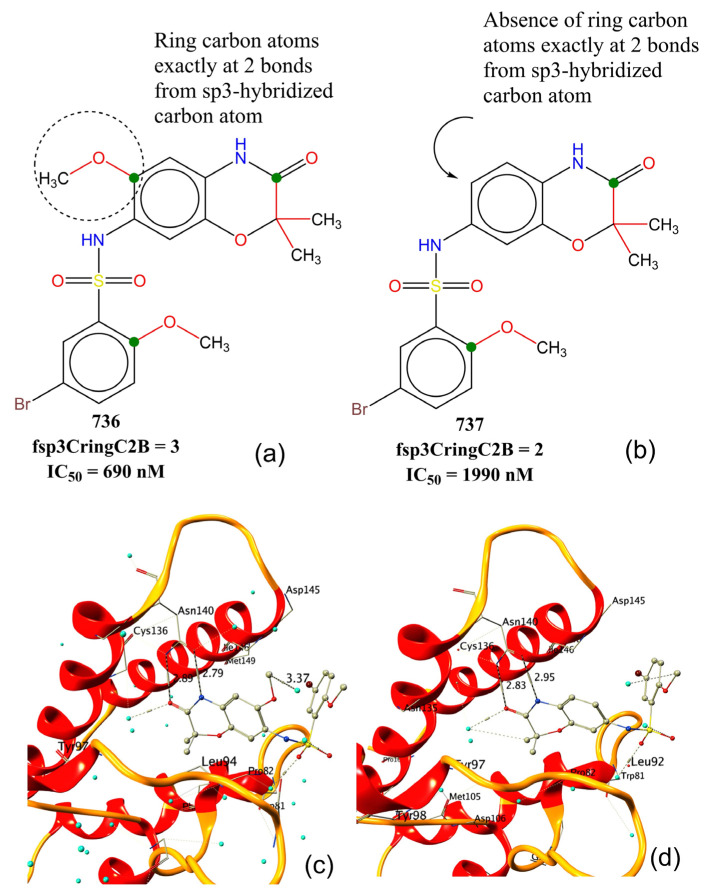
Comparison of BRD-4 inhibitory activity of molecule 736 with 737 with respect to fsp3CringC2B: (**a**,**b**) 2D representation of 736 and 737; (**c**,**d**) X-ray-resolved structures of 737 (pdb: 5z1r) and 736 (pdb: 5z1s). Cyan coloured spheres represent water molecules, and dashed lines signify interactions and distances in Å.

The molecular descriptor fsp3CringC2B represents the frequency of occurrence of ring carbon atoms exactly at two bonds from sp^3^-hybridised carbon atoms. If the same ring carbon atom was also present at less than two bonds from any other sp^3^-hybridised carbon atoms, then it was excluded while calculating fsp3CringC2B. Its positive coefficient in model-A and also a correlation of 0.30 with pIC_50_ indicate that increasing such a combination of ring and sp^3^-hybridised carbon atoms could lead to better inhibitory activities for BRD-4. For example, a comparison of molecule 736 with 737 indicates the significant influence of ring carbon atoms (shown using green dots in Figure 4a,b) at exactly two bonds from sp^3^-hybridised carbon atoms. This is further supported by their reported X-ray resolved structures with BRD-4. Molecule 736 (pdb: 5z1s [47]) has an additional water-mediated interaction with receptors with a distance of 3.37 Å (see Figure 4c) due to the -OCH_3_ group present in the benzoxazinone ring. The same -OCH_3_ group is responsible for increasing the value of fsp3CringC2B for 736, but it is absent in 737 (pdb: 5z1r [47]). The difference in IC_50_ for the following pairs of molecules further support the influence of fsp3CringC2B on the activity profile: 255 with 499, 725 with 716, 231 with 240, and 89 with 105, to mention a few.

From this discussion, it appears that ring carbon atoms alone are important. However, replacing fsp3CringC2B by number of ring carbon atoms as a descriptor in model-A reduced the statistical performance from R^2^_tr_ = 0.76 to 0.73. In addition, the number of ring carbon atoms has a correlation of 0.27 with pIC_50_. Therefore, fsp3CringC2B is a better descriptor than the number of ring carbon atoms.

com_C_4A, which stands for total number of carbon atoms within 4 Å from centre of mass (com) of molecule, has a positive coefficient in model-A. Therefore, increasing the value of com_C_4A leads to better inhibitory activities. This observation is supported by the fact that it has a correlation of 0.404 with pIC_50_, and molecules with IC_50_ < 10 nM (29 molecules) possess a high value of com_C_4A. In addition, a simple comparison of the following pairs of the molecules strengthens this observation: 620 with 621, 720 with 710, 724 with 717, 526 with 518, and 691 with 692, and 595 with 596. At first glance, it looks as if com_C_4A is pointing out the importance of the number of carbon atoms. However, nC (number of carbon atoms) has a correlation of 0.29 with pIC_50_ and substituting com_C_4A by nC led to a decrease in statistical performance of model-A from R^2^_tr_ = 0.76 to 0.69. Therefore, com_C_4A is a better choice as a variable for model-A.

**Figure 5:**
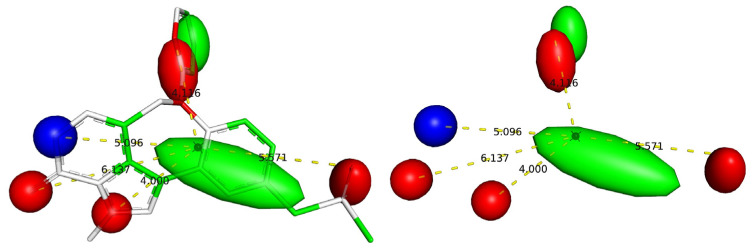
Pharmacophore model using most active molecule 207 (red: H-bond acceptor; blue: H-bond donor; green: hydrophobic region). Distances are shown using yellow dashed lines and figures indicate the distances in Å for different regions from center of mass. The black sphere represents the position of the center of mass of molecule.

As the presence of carbon is generally associated with the increased lipophilicity of a molecule, therefore, com_C_4A indicates that the lipophilic part must be concentrated near the com of the molecule for better activities. This in turn provides a crucial hint about the active site of BRD-4. It appears that a significant portion of the active site of BRD-4 is reasonably lipophilic in nature. This is supported by the fact that the active site of BRD-4 consists of a hydrophobic WPF shelf (see Figure 1) [11,18]. Thus, the findings of the present QSAR analysis are supported by the reported X-ray-resolved structure of BRD-4 enzyme. In addition, the pharmacophore model, depicted in Figure 5, generated using most active molecule 297, again points out the presence of a lipophilic region near the com of the molecule.

**Figure 6:**
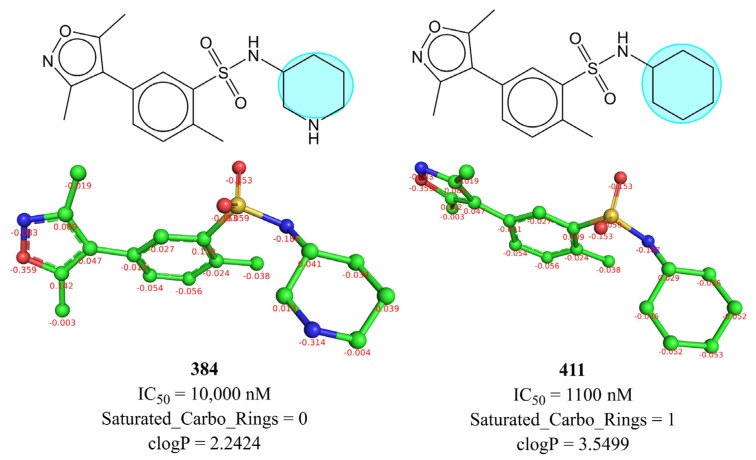
Depiction of influence of Saturated_Carbo_Rings on IC_50_ using molecule 384 and 411 as representative examples (2D and 3D representations with partial charges).

Another descriptor that also point outs the importance of lipophilicity of a molecule is flipoacc3B, which represents the frequency of occurrence of H-bond acceptor atoms exactly at three bonds from lipophilic atoms. However, an acceptor atom was excluded while calculating flipoacc3B if it is also present within two or less bonds from same or any other lipophilic atom. Evidently, the lipophilic part of a molecule close to H-bond acceptor moiety (O or N atoms) plays a crucial role in deciding the inhibitory effect for BRD-4. This is once again visible in the pharmacophore model depicted in Figure 6. A simple comparison of the following pairs of molecules supports this observation: 812 with 823, 7 with 8, and 4 with 10.

An easily interpretable and influential molecular descriptor is Saturated_Carbo_Rings, which corresponds to total number of saturated carbocyclic rings. It has positive coefficient in model-A; therefore, increasing such rings is beneficial. A comparison of IC_50_ for 411 with 384 (see Figure 6), 137 with 127, 73 with 67, 131 with 124, 60 with 61, 570 with 573, and 572, 230 with 247 is in favour of this observation.

The importance of Saturated_Carbo_Rings in model-A indicates that the lipophilicity and flexibility of a molecule are the actual factors governing an activity profile. It is noteworthy that clogP, which represents molecular lipophilicity, has a correlation of 0.193 with pIC_50_, whereas Saturated_Carbo_Rings has 0.240. Thus, Saturated_Carbo_Rings is a better choice, as it pinpoints the specific feature or part of the molecule (saturated carbocyclic rings), which is correlated with the activity due to its lipophilic nature, whereas clogP is a molecular property. A plausible reason could be the crucial role played by hydrophobic zones such as saturated carbocyclic rings at the periphery or outer part of molecule as a proxy for BRD4 selectivity through their interaction with the WPF shelf [6]. Therefore, saturated carbocyclic rings should be retained in future optimizations for better activity profiles. Thus, the present work is successful in identifying the significance of saturated carbocyclic rings as a novel unreported pharmacophore feature associated with BRD-4 inhibitory activity.

The molecular descriptor fsulfonSaroC8B (frequency of occurrence of aromatic carbon atoms exactly at eight bonds from sulphur atoms of Sulfone (-SO_2_-) group) has a positive coefficient in model-A. Consequently, increasing the value of fsulfonSaroC8B, favours binding with BRD-4. It is to be noted that if the same aromatic carbon atom is also present at ≤7 bonds from a sulphur atom of same or different Sulfone group through any path, then it was excluded while calculating fsulfonSaroC8B. Obviously, this descriptor signifies the importance of the Sulfone group (a highly polar group) and its correlation with aromatic rings (a lipophilic moiety) in deciding the binding with BRD-4. This is clearly reflected in the difference in the activity of the following pairs of molecules: 715 with 718, 723 with 724, 714 with 715, 707 with 716, 936 with 941, and 942 with 943. Recent studies indicate that Sulfone moiety is present in a cleft near the ZA-loop and establishes an H-bond with the -CONH- (amide) of backbone [19].

**Figure 7:**
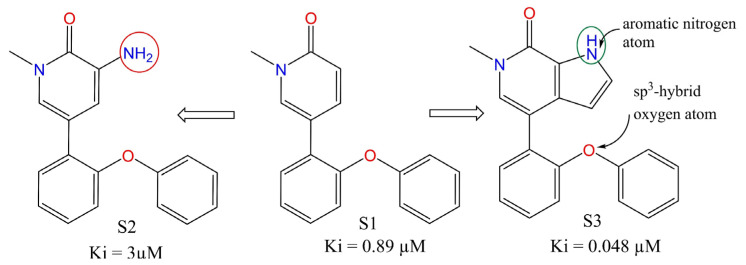
Effect of fsp3OaroN6B on BRD-4 inhibitory activity.

fsp3OaroN6B stands for the frequency of occurrence of aromatic nitrogen atoms exactly at six bonds from sp^3^-hybridised oxygen atom. If the same aromatic nitrogen atom is also present at ≤5 bonds from the same or any other sp^3^-hybridised oxygen atom through any path, then it was excluded while calculating fsp3OaroN6B. For example, 79 with 609, 81 with 620, and 614 with 615, to mention a few. In our previous study [20], we identified a similar descriptor notringO_acc_6B (total number of all non-ring Oxygen atoms present within a distance of six bonds from H-bond acceptor atoms) as an important pharmacophoric feature that governs the binding affinity (Ki) of a molecule for BRD-4. Thus, a consensus between the previous and the present study indicates that a molecule must have an H-bond acceptor (preferably aromatic nitrogen) at a distance of six bonds from a sp^3^-hybridised oxygen atom (non-ring oxygen favoured). This observation is supported by the difference in the activity of molecule S1, S2, and S3 [48] (see Figure 7). To add further, the sp^3^-hybridised oxygen atom is present as a linker between two aromatic rings or as an -OR (alkoxy) group in a good number of molecules [20].

The only descriptor with a negative coefficient in model-A is fplaNN4B, which corresponds to frequency of occurrence of nitrogen atoms exactly at 4 bonds from planer nitrogen atoms. If the same nitrogen atom is also present at ≤3 bonds from the same or any other planer nitrogen atom through any path, then it was excluded while calculating fplaNN4B. The following pairs of molecules have a significant difference in their activities, which could be attributed to the presence of fplaNN4B as a structural feature: 945 with 954, 936 with 944, 411 with 385, 170 with 171, 722 with 714, 563 with 577, 737 with 734, 239 with 240. Therefore, such a combination of nitrogen atoms should be avoided to have better inhibition of BRD-4.

## 4. Materials and Methods

The present work follows the standard procedure recommended by OECD and different researchers to perform QSAR analysis [49,50,51]. All the software were used with default settings to obtain a QSAR model possessing a balance of predictive ability and mechanistic interpretation; however, some settings were changed, which have been reported at appropriate places. The different steps are as follows.

**Figure 8:**
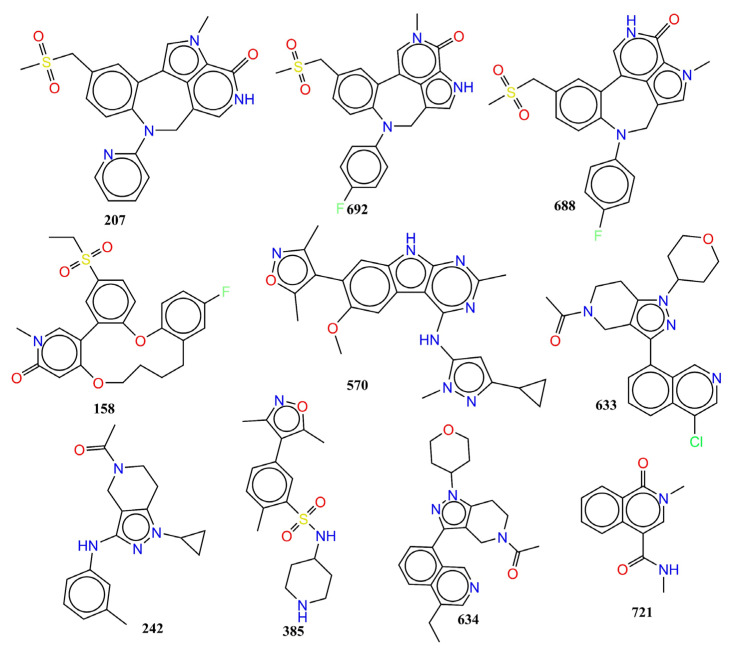
Representative examples to indicate the structural scaffold diversity in the present dataset.

**Step-1:** Collection of data and curation: The present work commenced with the collection of a large dataset of 2026 experimentally tested BRD-4 inhibitors from a free and publicly available database BindingDB (https://www.bindingdb.org/bind/index.jsp, accessed on 16 March 2022). A QSAR analysis is significantly influenced by the quality of data, its composition, and its appropriate curation before further processing [50,52,53,54]. Therefore, in the next step, data curation was performed [55], which involved the removal of duplicate entries, organometallic compounds, salts, molecules with ambiguous IC_50_ values, etc. This reduced the dataset to 980 molecules only. The reduced dataset still consists of molecules with experimental IC_50_ (nM) in the range 1 nM to 15 µM and the presence of diverse scaffolds such as heterocyclic rings, positional isomers, stereoisomers, etc., enhancing the chemical space and consequently widening the applicability of the newly developed model. The SMILES (Simplified Molecular Input Line Entry System) notations, including experimental IC_50_ and pIC_50_ (=−log_10_IC_50_) of all the molecules used in the present work, are available in Supplementary Materials. For the sake of convenience, representative examples have been presented in Figure 8 to depict the structural diversity of the current dataset.

In Table 2, five most and least active molecules have been included as examples only along with their SMILES notation: IC_50_ (nM) and pIC_50_ (M).

**Step-2:** In the next step, SMILES notations were used to develop the optimised 3D structures (semi-empirical PM3 method) of the molecules, accomplished using OpenBabel 2.4 [56] and MOPAC 2012 (openmopac.net) using default settings.

**Step-3:** A QSAR model achieves a balance of mechanistic interpretation and predictive ability if a good number of diverse molecular descriptors are calculated, followed by adequate pruning to reduce the chances of overfitting from noisy redundant descriptors [57]. The next step involved the calculation of myriad number of 1D- to 3D-molecular descriptors for all molecules. For this, *PyDescriptor* [45] and DataWarrior [46] were used, which generated more than 40,000 molecular descriptors for a single molecule. Obviously, the descriptor pool contained a good number of redundant molecular descriptors; therefore, highly correlated (|R| > 0.95) and nearly constant (>98%) variables were removed using QSARINS 2.2.4 [58]. This considerably decreased the size of set of molecular descriptor pool from 30,000 to 4326, which still contained a variety of molecular descriptors.

### 4.1. Splitting the Data Set into Training and External Sets and Subjective Feature Selection (SFS)

**Figure 9:**
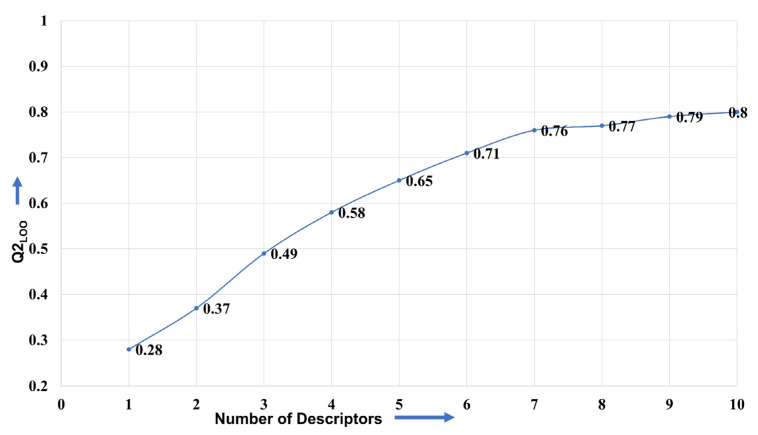
Graph between number of descriptors against leave-one-out coefficient of determination Q^2^_LOO_ to identify the optimum number of descriptors.

For developing a useful QSAR model and its appropriate validation, it is essential to divide the dataset into training and external (also called as prediction or test set) sets [50,52,53,54,59]. Consequently, in the present work, the dataset was randomly divided into training (80% = 785 molecules) and external (20% = 195 molecules) sets to minimise any bias. The only purpose of the training set was to choose suitable number of variables (molecular descriptors), whereas the external set was employed only for validation purpose, i.e., external validation of the model (Predictive QSAR). Genetic Algorithm (GA) and multi-linear regression (MLR) available in QSARINS 2.2.4 were used for model building. For this, Q^2^_LOO_ was used as a fitness function, and the number of generations was set to 10,000. A decisive step in QSAR modelling is to select the optimum number of molecular descriptors for model building to avoid over-fitting and to obtain acceptable interpretability. Consequently, the heuristic search involved building multiple models from univariate to multivariate with the successive addition of molecular descriptors until there was an increase in the value of Q^2^_LOO_, which is called the breaking point [39,60]. A 2D graph between the number of molecular descriptors involved in the models and Q^2^_LOO_ values has been depicted in Figure 9. The number of variables matching with the breaking point was considered optimal for model building as there was no improvement in the statistical performance of model upon the inclusion of additional molecular descriptors. The analysis led to the matching of breaking points with seven variables. Therefore, QSAR models with more than seven descriptors were excluded.

### 4.2. Building Regression Model and Its Validation

Appropriate validations involving cross/inter validation, external validation, Y-randomization analysis, and applicability domain (Williams plot) are necessary to estimate the reliability and general applicability of a QSAR model [25,31,33,50,61]. A properly validated QSAR model finds its usage for QSAR-based virtual screening, lead/hit optimization, decision making, etc. The following validation parameters and their recommended threshold values are usually used to assess a model [39,60]: R^2^_tr_ (coefficient of determination) ≥ 0.6; Q^2^_loo_ (cross-validated coefficient of determination for leave-one-out) ≥ 0.5; Q^2^_LMO_ (cross-validated coefficient of determination for leave-many-out) ≥ 0.6, R^2^ > Q^2^; R^2^_ex_ (external coefficient of determination) ≥ 0.6; CCC (Concordance Correlation Coefficient) ≥ 0.80; Q^2^-F^n^ ≥ 0.60; high values of external validation parameters R^2^_ex_, Q^2^_F1_, Q^2^_F2_, and Q^2^_F3_, with low values of R^2^_Yscr_ (coefficient of determination for Y-randomization); RMSE (Root mean square error); MAE (Mean absolute error); RMSE_tr_ < RMSE_cv_. The formulae for calculating these statistical parameters are available in Supplementary Materials. In the present analysis, a Williams plot was used to assess the applicability domain of the newly developed QSAR model.

### 4.3. Pharmacophore Model

For pharmacophore modelling, the 3D-optimised structure of the most active molecule, 207, was selected. The model was generated using LIQUID [62,63], a free and easy to use PyMOL plugin, using default settings, except that the contour region was set to 3 for H-bond donor/acceptor and hydrophobic regions.

### 4.4. Other Experimental Details

The reported X-ray resolved structures (pdb 5z1r and 5z1s) were downloaded from Protein Data Bank (www.rcsb.org accessed on 13 April 2022). PyMOL version 2.4 has been used for the depiction of molecular interactions between the compounds and the protein.

## 5. Conclusions

In the present study, a seven-descriptor-based and rigorously validated GA–MLR QSAR model with R^2^_tr_ = 0.79, Q^2^_LMO_ = 0.79, and R^2^_ex_ = 0.78 was derived to identify the significant pharmacophoric features that influence BRD-4 inhibitory activity. As mentioned earlier, it is essential to perceive salient and visually unrecognizable pharmacophoric features linked with BRD-4 inhibitory activity for different chemical scaffolds. The analysis indicates that the presence of ring carbon and nitrogen atoms, occurrence of carbon atoms near the center of mass of a molecule, specific combination of planer nitrogen with ring carbon, donor and acceptor atoms, etc., are prominent features to be retained in future optimizations. On the other hand, a combination of nitrogen atoms with planer nitrogen atoms exactly at four bonds should be avoided for better BRD-4 inhibitory activity. The reported crystal structures of BRD-4 inhibitors strengthen these observations. The present study efficaciously captured and reported novel pharmacophoric features and has a good balance of predictive ability and mechanistic interpretations.

## Figures and Tables

**Table 1:** Details of molecular descriptors present in model-A.

Variable	Description	Software Used for Calculation
fsp3CringC2B	Frequency of occurrence of ring carbon atoms exactly at 2 bonds from sp^3^-hybridised carbon atoms	PyDescriptor [45]
com_C_4A	Total number of carbon atoms within 4 Å from centre of mass (com) of molecule	PyDescriptor
Saturated_Carbo_Rings	Total number of saturated rings containing carbon atoms only	DataWarrior [46]
fsulfonSaroC8B	Frequency of occurrence of aromatic carbon atoms exactly at 8 bonds from sulphur atoms of Sulfone group	PyDescriptor
fsp3OaroN6B	Frequency of occurrence of aromatic nitrogen atoms exactly at 6 bonds from sp^3^-hybridised oxygen atoms	PyDescriptor
flipoacc3B	Frequency of occurrence of H-bond acceptor atoms exactly at 3 bonds from lipophilic atoms	PyDescriptor
fplaNN4B	Frequency of occurrence of nitrogen atoms exactly at 4 bonds from planer nitrogen atoms	PyDescriptor

**Table 2:** SMILES notation and IC_50_ (nM) and pIC_50_ (M) of five most and least active molecules of the selected data set.

SN	Ligand SMILES	IC_50_ (nM)	pIC_50_ (M)
207	Cn1cc2-c3cc(CS(C)(=O)=O)ccc3N(Cc3c[nH]c(=O)c1c23)c1ccccn1	1	9
692	Cn1cc2-c3cc(CS(C)(=O)=O)ccc3N(Cc3c[nH]c(c23)c1=O)c1ccc(F)cc1	1.5	8.824
158	CCS(=O)(=O)c1ccc2Oc3ccc(F)cc3CCCCOc3cc(=O)n(C)cc3-c2c1	2	8.699
570	COc1cc2c(cc1-c1c(C)noc1C)[nH]c1nc(C)nc(Nc3cc(nn3C)C3CC3)c21	2	8.699
688	Cn1cc2CN(c3ccc(F)cc3)c3ccc(CS(C)(=O)=O)cc3-c3c[nH]c(=O)c1c23	2.5	8.602
633	CC(=O)N1CCc2c(C1)c(nn2C1CCOCC1)-c1cccc2c(Cl)cncc12	14,000	4.854
242	CC(=O)N1CCc2c(C1)c(Nc1cccc(C)c1)nn2C1CC1	15,000	4.824
385	Cc1noc(C)c1-c1ccc(C)c(c1)S(=O)(=O)NC1CCNCC1	15,000	4.824
634	CCc1cncc2c(cccc12)-c1nn(C2CCOCC2)c2CCN(Cc12)C(C)=O	15,000	4.824
721	CNC(=O)c1cn(C)c(=O)c2ccccc12	15,000	4.824

## Data Availability

Data is contained within the article and Supplementary Material.

## Supplementary Materials

The following supporting information can be downloaded at: https://www.mdpi.com/article/10.3390/ph15060745/s1.

## Abbreviations

SMILES	Simplified molecular-input line-entry system
GA	Genetic algorithm
MLR	Multiple linear regression
QSAR	Quantitative structure−activity relationship
WHO	World Health Organization
OLS	Ordinary least square
QSARINS	QSAR Insubria
OECD	Organisation for Economic Co-operation and Development

## References

[1] Boer R.A., Meijers W.C., Meer P., Veldhuisen D.J. (2019). Cancer and heart disease: Associations and relations. Eur. J. Heart Fail..

[2] Fidanze S.D., Liu D., Mantei R.A., Hasvold L.A., Pratt J.K., Sheppard G.S., Wang L., Holms J.H., Dai Y., Aguirre A. (2018). Discovery and optimization of novel constrained pyrrolopyridone BET family inhibitors. Bioorg. Med. Chem. Lett..

[3] Guest E.E., Pickett S.D., Hirst J.D. (2021). Structural variation of protein–ligand complexes of the first bromodomain of BRD4. Org. Biomol. Chem..

[4] Liu Z., Chen H., Wang P., Li Y., Wold E.A., Leonard P.G., Joseph S., Brasier A.R., Tian B., Zhou J. (2020). Discovery of Orally Bioavailable Chromone Derivatives as Potent and Selective BRD4 Inhibitors: Scaffold Hopping, Optimization, and Pharmacological Evaluation. J. Med. Chem..

[5] Alqahtani A., Choucair K., Ashraf M., Hammouda D.M., Alloghbi A., Khan T., Senzer N., Nemunaitis J. (2019). Bromodomain and extra-terminal motif inhibitors: A review of preclinical and clinical advances in cancer therapy. Future Sci. OA.

[6] Speck-Planche A., Scotti M.T. (2018). BET bromodomain inhibitors: Fragment-based in silico design using multi-target QSAR models. Mol. Divers.

[7] Duan Y., Guan Y., Qin W., Zhai X., Yu B., Liu H. (2018). Targeting Brd4 for cancer therapy: Inhibitors and degraders. MedChemComm.

[8] Zhao Y., Bai L., Liu L., McEachern D., Stuckey J.A., Meagher J.L., Yang C.-Y., Ran X., Zhou B., Hu Y. (2017). Structure-Based Discovery of 4-(6-Methoxy-2-methyl-4-(quinolin-4-yl)-9*H*-pyrimido[4,5-*b*]indol-7-yl)-3,5-dimethylisoxazole (CD161) as a Potent and Orally Bioavailable BET Bromodomain Inhibitor. J. Med. Chem..

[9] Xing J., Lu W., Liu R., Wang Y., Xie Y., Zhang H., Shi Z., Jiang H., Liu Y.-C., Chen K. (2017). Machine-Learning-Assisted Approach for Discovering Novel Inhibitors Targeting Bromodomain-Containing Protein 4. J. Chem. Inf. Model..

[10] Kuang M., Zhou J., Wang L., Liu Z., Guo J., Wu R. (2015). Binding Kinetics versus Affinities in BRD4 Inhibition. J. Chem. Inf. Model..

[11] Ember S.W.J., Zhu J.-Y., Olesen S.H., Martin M.P., Becker A., Berndt N., Georg G.I., Schönbrunn E. (2014). Acetyl-lysine Binding Site of Bromodomain-Containing Protein 4 (BRD4) Interacts with Diverse Kinase Inhibitors. ACS Chem. Biol..

[12] Shorstova T., Foulkes W.D., Witcher M. (2021). Achieving clinical success with BET inhibitors as anti-cancer agents. Br. J. Cancer.

[13] Wang L., Pratt J.K., Soltwedel T., Sheppard G.S., Fidanze S.D., Liu D., Hasvold L.A., Mantei R.A., Holms J.H., McClellan W.J. (2017). Fragment-Based, Structure-Enabled Discovery of Novel Pyridones and Pyridone Macrocycles as Potent Bromodomain and Extra-Terminal Domain (BET) Family Bromodomain Inhibitors. J. Med. Chem..

[14] Filippakopoulos P., Qi J., Picaud S., Shen Y., Smith W.B., Fedorov O., Morse E.M., Keates T., Hickman T.T., Felletar I. (2010). Selective inhibition of BET bromodomains. Nature.

[15] Liu Z., Wang P., Chen H., Wold E.A., Tian B., Brasier A.R., Zhou J. (2017). Drug Discovery Targeting Bromodomain-Containing Protein 4. J. Med. Chem..

[16] Filippakopoulos P., Picaud S., Mangos M., Keates T., Lambert J.-P., Barsyte-Lovejoy D., Felletar I., Volkmer R., Müller S., Pawson T. (2012). Histone Recognition and Large-Scale Structural Analysis of the Human Bromodomain Family. Cell.

[17] Donati B., Lorenzini E., Ciarrocchi A. (2018). BRD4 and Cancer: Going beyond transcriptional regulation. Mol. Cancer.

[18] Zaware N., Zhou M.-M. (2019). Bromodomain biology and drug discovery. Nat. Struct. Mol. Biol..

[19] Sheppard G.S., Wang L., Fidanze S.D., Hasvold L.A., Liu D., Pratt J.K., Park C.H., Longenecker K., Qiu W., Torrent M. (2020). Discovery of *N*-Ethyl-4-[2-(4-fluoro-2,6-dimethyl-phenoxy)-5-(1-hydroxy-1-methyl-ethyl)phenyl]-6-methyl-7-oxo-1*H*-pyrrolo[2,3-*c*]pyridine-2-carboxamide (ABBV-744), a BET Bromodomain Inhibitor with Selectivity for the Second Bromodomain. J. Med. Chem..

[20] Masand V.H., Patil M.K., El-Sayed N.N.E., Zaki M.E.A., Almarhoon Z., Al-Hussain S.A. (2021). Balanced QSAR analysis to identify the structural requirements of ABBV-075 (Mivebresib) analogues as bromodomain and extraterminal domain (BET) family bromodomain inhibitor. J. Mol. Struct..

[21] Tahir A., Alharthy R.D., Naseem S., Mahmood N., Ahmed M., Shahzad K., Akhtar M.N., Hameed A., Sadiq I., Nawaz H. (2018). Investigations of Structural Requirements for BRD4 Inhibitors through Ligand- and Structure-Based 3D QSAR Approaches. Molecules.

[22] Tong J.-B., Luo D., Feng Y., Bian S., Zhang X., Wang T.-H. (2021). Structural modification of 4, 5-dihydro-[1, 2, 4] triazolo [4, 3-f] pteridine derivatives as BRD4 inhibitors using 2D/3D-QSAR and molecular docking analysis. Mol. Divers..

[23] Babatunde Samuel Obadawo O.E.O. (2019). Mayowa Monday Anifowose, Kehinde Henry Fagbohungbe, Justinah Solayide Amoko, QSAR modeling of novel substituted 4-Phenylisoquinolinones as potent BET bromodomain (BRD4-BD1) inhibitors. Biomed. Lett..

[24] Zaki M.E.A., Al-Hussain S.A., Masand V.H., Akasapu S., Lewaa I. (2021). QSAR and Pharmacophore Modeling of Nitrogen Heterocycles as Potent Human N-Myristoyltransferase (Hs-NMT) Inhibitors. Molecules.

[25] Gramatica P. (2020). Principles of QSAR Modeling. Int. J. Quant. Struct. Prop. Relatsh..

[26] Polishchuk P. (2017). Interpretation of Quantitative Structure–Activity Relationship Models: Past, Present, and Future. J. Chem. Inf. Model..

[27] Fujita T., Winkler D.A. (2016). Understanding the Roles of the “Two QSARs”. J. Chem. Inf. Model..

[28] Krstajic D., Buturovic L.J., Leahy D.E., Thomas S. (2014). Cross-validation pitfalls when selecting and assessing regression and classification models. J. Cheminform..

[29] Gramatica P. (2014). External Evaluation of QSAR Models, in Addition to Cross-Validation Verification of Predictive Capability on Totally New Chemicals. Mol. Inf..

[30] Gütlein M., Helma C., Karwath A., Kramer S. (2013). A Large-Scale Empirical Evaluation of Cross-Validation and External Test Set Validation in (Q)SAR. Mol. Inf..

[31] Gramatica P. (2013). On the development and validation of QSAR models. Methods Mol. Biol..

[32] Chirico N., Gramatica P. (2012). Real external predictivity of QSAR models. Part 2. New intercomparable thresholds for different validation criteria and the need for scatter plot inspection. J. Chem. Inf. Model..

[33] Chirico N., Gramatica P. (2011). Real external predictivity of QSAR models: How to evaluate it? Comparison of different validation criteria and proposal of using the concordance correlation coefficient. J. Chem. Inf. Model..

[34] Consonni V., Ballabio D., Todeschini R. (2009). Comments on the definition of the Q2 parameter for QSAR validation. J. Chem. Inf. Model..

[35] Rao R.B., Fung G., Rosales R. (2008). On the Dangers of Cross-Validation. An Experimental Evaluation.

[36] Gramatica P., Giani E., Papa E. (2007). Statistical external validation and consensus modeling: A QSPR case study for Koc prediction. J. Mol. Graph. Model..

[37] Hawkins D.M., Basak S.C., Mills D. (2003). Assessing model fit by cross-validation. J. Chem. Inf. Comput. Sci..

[38] Masand V.H., Mahajan D.T., Nazeruddin G.M., Hadda T.B., Rastija V., Alfeefy A.M. (2015). Effect of information leakage and method of splitting (rational and random) on external predictive ability and behavior of different statistical parameters of QSAR model. Med. Chem. Res..

[39] Zaki M.E.A., Al-Hussain S.A., Bukhari S.N.A., Masand V.H., Rathore M.M., Thakur S.D., Patil V.M. (2022). Exploring the Prominent and Concealed Inhibitory Features for Cytoplasmic Isoforms of Hsp90 Using QSAR Analysis. Pharmaceuticals.

[40] Kar S., Roy K., Leszczynski J. (2018). Applicability Domain: A Step Toward Confident Predictions and Decidability for QSAR Modeling. Computational Toxicology.

[41] Gramatica P., Kovarich S., Roy P.P. (2013). Reply to the comment of *S. Rayne* on “QSAR model reproducibility and applicability: A case study of rate constants of hydroxyl radical reaction models applied to polybrominated diphenyl ethers and (benzo-)triazoles”. J. Comput. Chem..

[42] Roy P.P., Kovarich S., Gramatica P. (2011). QSAR model reproducibility and applicability: A case study of rate constants of hydroxyl radical reaction models applied to polybrominated diphenyl ethers and (benzo-)triazoles. J. Comput. Chem..

[43] Gadaleta D., Mangiatordi G.F., Catto M., Carotti A., Nicolotti O. (2016). Applicability Domain for QSAR Models. Int. J. Quant. Struct. -Prop. Relatsh..

[44] Tropsha A., Golbraikh A. (2007). Predictive QSAR modeling workflow, model applicability domains, and virtual screening. Curr. Pharm. Des..

[45] Masand V.H., Rastija V. (2017). PyDescriptor: A new PyMOL plugin for calculating thousands of easily understandable molecular descriptors. Chemom. Intell. Lab. Syst..

[46] Sander T., Freyss J., von Korff M., Rufener C. (2015). DataWarrior: An Open-Source Program For Chemistry Aware Data Visualization And Analysis. J. Chem. Inf. Model..

[47] Xiang Q., Zhang Y., Li J., Xue X., Wang C., Song M., Zhang C., Wang R., Li C., Wu C. (2018). Y08060: A Selective BET Inhibitor for Treatment of Prostate Cancer. ACS Med. Chem. Lett..

[48] McDaniel K.F., Wang L., Soltwedel T., Fidanze S.D., Hasvold L.A., Liu D., Mantei R.A., Pratt J.K., Sheppard G.S., Bui M.H. (2017). Discovery of *N*-(4-(2,4-Difluorophenoxy)-3-(6-methyl-7-oxo-6,7-dihydro-1*H*-pyrrolo[2,3-*c*]pyridin-4-yl)phenyl)ethanesulfonamide (ABBV-075/Mivebresib), a Potent and Orally Available Bromodomain and Extraterminal Domain (BET) Family Bromodomain Inhibitor. J. Med. Chem..

[49] Dearden J.C., Cronin M.T., Kaiser K.L. (2009). How not to develop a quantitative structure-activity or structure-property relationship (QSAR/QSPR). SAR QSAR Environ. Res..

[50] Cherkasov A., Muratov E.N., Fourches D., Varnek A., Baskin I.I., Cronin M., Dearden J., Gramatica P., Martin Y.C., Todeschini R. (2014). QSAR modeling: Where have you been? Where are you going to?. J. Med. Chem..

[51] Huang J., Fan X. (2011). Why QSAR fails: An empirical evaluation using conventional computational approach. Mol. Pharm..

[52] Muratov E.N., Bajorath J., Sheridan R.P., Tetko I.V., Filimonov D., Poroikov V., Oprea T.I., Baskin I.I., Varnek A., Roitberg A. (2020). QSAR without borders. Chem. Soc. Rev..

[53] Golbraikh A., Muratov E., Fourches D., Tropsha A. (2014). Data set modelability by QSAR. J. Chem. Inf. Model..

[54] Martin T.M., Harten P., Young D.M., Muratov E.N., Golbraikh A., Zhu H., Tropsha A. (2012). Does rational selection of training and test sets improve the outcome of QSAR modeling?. J. Chem. Inf. Model..

[55] Fourches D., Muratov E., Tropsha A. (2010). Trust, but verify: On the importance of chemical structure curation in cheminformatics and QSAR modeling research. J. Chem. Inf. Model..

[56] O’Boyle N.M., Banck M., James C.A., Morley C., Vandermeersch T., Hutchison G.R. (2011). Open Babel: An open chemical toolbox. J. Cheminform..

[57] Tetko I.V., Sushko I., Pandey A.K., Zhu H., Tropsha A., Papa E., Oberg T., Todeschini R., Fourches D., Varnek A. (2008). Critical assessment of QSAR models of environmental toxicity against Tetrahymena pyriformis: Focusing on applicability domain and overfitting by variable selection. J. Chem. Inf. Model..

[58] Gramatica P., Chirico N., Papa E., Cassani S., Kovarich S. (2013). QSARINS: A new software for the development, analysis, and validation of QSAR MLR models. J. Comput. Chem..

[59] Tropsha A., Gramatica P., Gombar V.K. (2003). The Importance of Being Earnest Validation is the Absolute Essential for Successful Application and Interpretation of QSPR Models. QSAR Comb. Sci..

[60] Zaki M.E.A., Al-Hussain S.A., Masand V.H., Sabnani M.K., Samad A. (2021). Mechanistic and Predictive QSAR Analysis of Diverse Molecules to Capture Salient and Hidden Pharmacophores for Anti-Thrombotic Activity. Int. J. Mol. Sci..

[61] Gramatica P. (2007). Principles of QSAR models validation internal and external. QSAR Comb. Sci..

[62] Yuan S., Chan H.C.S., Hu Z. (2017). Using PyMOL as a platform for computational drug design. WIREs Comput. Mol. Sci..

[63] Tanrikulu Y., Nietert M., Scheffer U., Proschak E., Grabowski K., Schneider P., Weidlich M., Karas M., Gobel M., Schneider G. (2007). Scaffold hopping by “fuzzy” pharmacophores and its application to RNA targets. Chembiochem.

