# Supplementary material for: Mechanistic Analysis of Chemically Diverse Bromodomain-4 Inhibitors Using Balanced QSAR Analysis and Supported by X-ray Resolved Crystal Structures
Source: Pharmaceuticals (Basel). 2022 Jun 14;15(6):745. doi: 10.3390/ph15060745 (PMC9231298; doi:10.3390/ph15060745)
Supplement: Supplementary file 1 [file pharmaceuticals-15-00745-s001.zip › Supplementary-material.pdf]

Mechanistic analysis of chemically diverse Bromodomain-4 inhibitors using balanced QSAR analysis and supported by X-ray resolved crystal structures

Magdi E.A. Zaki[1*], Sami A. Al-Hussain[1], Aamal A. Al-Mutairi[1], Vijay H. Masand[2*], Abdul Samad[3], Rahul D. Jawarkar[4]

[1] Department of Chemistry, Faculty of Science, Imam Mohammad Ibn Saud Islamic University, Riyadh 13318, Saudi Arabia (Mezaki@imamu.edu.sa, and sahussain@imamu.edu.sa, aamutairi@imamu.edu.sa)
[2] Department of Chemistry, Vidya Bharati Mahavidyalaya, Amravati, Maharashtra, India- 444 601
[3] Department of Pharmaceutical Chemistry, Faculty of Pharmacy, Tishk International University, Erbil, Kurdistan Region, Iraq
[4] Department of Medicinal Chemistry, Dr. Rajendra Gode Institute of Pharmacy, University-Mardi Road, Amravati, Maharashtra, India
* Correspondence: vijaymasand@gmail.com, Mezaki@imamu.edu.sa

Validation parameters associated with Model-A:

R^2_{tr} = 0.762, $R^2_{adj.}$ = 0.76, R^2_{tr} - $R^2_{adj.}$ = 0.002, *LOF* = 0.157, *Kxx* = 0.154, *ΔK* = 0.096, $RMSE_{tr}$ = 0.389, MAE_{tr} = 0.326, RSS_{tr} = 118.973, CCC_{tr} = 0.865, *s* = 0.391, *F* = 355.446, R^2_{cv} $(Q^2 loo)$ = 0.757, R^2-R^2_{cv} = 0.005, $RMSE_{cv}$ = 0.393, MAE_{cv} = 0.329, $PRESS_{cv}$ = 121.434, CCC_{cv} = 0.862, Q^2_{LMO} = 0.756, R^2_{Yscr} = 0.009, Q^2_{Yscr} = -0.012, $RMSE_{ex}$ = 0.392, MAE_{ex} = 0.323, $PRESS_{ext}$ = 29.999, R^2_{ex} = 0.762, Q^2-F^1 = 0.762, Q^2-F^2 = 0.76, Q^2-F^3 = 0.758, CCC_{ex} = 0.86, R^2-*ExPy* = 0.762, R'^2_o = 0.661, *k'* = 0.998, $1-(R^2/R'^2_o)$ = 0.132, r'^2m = 0.52, R_o^2 = 0.761, *k* = 0.998, $1-(R^2\text{-}ExPy/R_o^2)$ = 0.002, r^2m = 0.736

Statistical symbols with names and explanations:

R^2 – correlation coefficient, Q^2 – leave-one-out 'crossvalidated R^2', R^2_{adj} - adjusted R^2, SEE – standard error of estimates, RMSE - root mean squared error, MAE - mean absolute error, CCC - concordance correlation coefficient, for the training (tr), and test (ex) sets; R^2_{LMO} and Q^2_{LMO} – leave many-out correlation coefficient and cross-validation coefficients; R^2_{Yrand} and Q^2_{Yrand} – Y- scramble correlation and cross-validation coefficients;

Statistical parameters for used for validation of QSAR models:

$$R^2 = 1 - \frac{\sum(y_i - \hat{y}_i)^2}{\sum(y_i - \bar{y})^2}$$

where y_i are the observed values of the response, ȳ the corresponding average, ŷ are the calculated values

$$Q^2 = 1 - \frac{\sum(y_i - \hat{y}_i)^2}{\sum(y_i - \bar{y})^2}$$

where y_i are the observed values of the response, ȳ the corresponding average, ŷ are the values predicted for each object when it is not in the training set.

$$Q^2_{F1} = 1 - \frac{\sum_{i=1}^{n_{EXT}}(y_i - \hat{y}_i)^2}{\sum_{i=1}^{n_{EXT}}(y_i - \bar{y}_{TR})^2}$$

where y_i are the observed values of the response, ȳ the corresponding average, ŷ are the calculated values

$$Q^2_{F2} = 1 - \frac{\sum_{i=1}^{n_{EXT}}(y_i - \hat{y}_i)^2}{\sum_{i=1}^{n_{EXT}}(y_i - \bar{y}_{EXT})^2}$$

where y_i are the observed values of the response, ȳ the corresponding average, ŷ are the calculated values

$$Q^2_{F3} = 1 - \frac{\left[\sum_{i=1}^{n_{EXT}}(y_i - \hat{y}_i)^2\right]/n_{EXT}}{\left[\sum_{i=1}^{n_{TR}}(y_i - \bar{y}_{TR})^2\right]/n_{TR}}$$

where y_i are the observed values of the response, ȳ the corresponding average, ŷ are the calculated values

$$CCC = \frac{2\sum_{i=1}^{n_{EXT}}(y_i - \bar{y})(\hat{y}_i - \bar{\hat{y}})}{\sum_{i=1}^{n_{EXT}}(y_i - \bar{y})^2 + \sum_{i=1}^{n_{EXT}}(\hat{y}_i - \bar{\hat{y}})^2 + n_{EXT}(\bar{y} - \bar{\hat{y}})^2}$$

$$k = \frac{\sum_{i=i}^{n_{EXT}} y_i \hat{y}_i}{\sum_{i=1}^{n_{EXT}} \hat{y}_i^2}$$

$$k' = \frac{\sum_{i=i}^{n_{EXT}} y_i \hat{y}_i}{\sum_{i=1}^{n_{EXT}} y_i^2}$$

$$r_m^2 = r^2 \left(1 - \sqrt{r^2 - r_0^2}\right)$$

$$\overline{r_m^2} = \frac{(r_m^2 + r_m'^2)}{2}$$

$$RMSE = \sqrt{\frac{\sum_{i=1}^{n_{EXT}} (y_i - \hat{y}_i)^2}{n_{EXT}}}$$

$$MAE = \frac{\sum_{i=1}^{n_{EXT}} |y_i - \hat{y}_i|}{n_{EXT}}$$

where y_i are the observed values of the response, $\bar{y}$ the corresponding average, $\hat{y}$ are the calculated values
